# *Carex ensiformis* (*C.* subg. *Siderosticta*), a new species from southern China based on morphological and molecular evidence

**DOI:** 10.1186/s40529-026-00498-y

**Published:** 2026-10-09

**Authors:** Zhaocen Lu, Yifei Lu, Xiaofeng Jin

**Affiliations:** 1 College of Forestry (College of Life Sciences), Zhejiang A&F University, Hangzhou, Zhejiang 311300 China; 2 Guangxi Key Laboratory of Plant Conservation and Restoration Ecology in Karst Terrain, Guangxi Institute of Botany, Guangxi Zhuang Autonomous Region and Chinese Academy of Sciences, Guilin, Guangxi 541006 China

**Keywords:** *Carex* subg. *Siderosticta*, Cyperaceae, Morphology, Micromorphology, New species, Taxonomy

## Abstract

**Background:**

*Carex *subg. *Siderosticta *is one of the six subgenera recognized in the recently revised infrageneric classification of *Carex *based on phylogenetic studies. It represents the earliest-diverging lineage within *Carex *and it is sister to all remaining species in the genus. This morphologically diverse subgenus is distributed across East and Southeast Asia. Historically, the approximately 30 species of this subgenus were assigned to four morphology-based sections, namely *Siderostictae*, *Hemiscaposae*, *Surculosae*, and *Hypolytroides*.

**Results:**

Phylogenetic analyses based on three plastid (*matK*, *ndhF*, and *rps16*) and two nuclear (ETS and ITS) DNA markers recovered the four individuals of *Carex ensiformis *as a strongly supported monophyletic lineage. Micromorphological observations revealed that *C. ensiformis *differs from the morphologically similar species *C. damiaoshanensis *in the slightly undulate anticlinal cell walls of the nutlet epidermis and a markedly shorter beak at the nutlet apex. Morphologically, the new species can be distinguished from *C. damiaoshanensis *by its more numerous inflorescence branches and spikes, as well as smaller utricles, nutlets, and shorter glumes.

**Conclusions:**

Both morphological and molecular evidence reveal that *Carex ensiformis* is a new species belonging to subg. *Siderosticta* from southern China. The discovery of this species enriches our understanding of species diversity and taxonomic complexity within the subgenus, and emphasizes the importance of continued taxonomic studies in this part of Asia.

## Introduction

*Carex* L., comprising more than 2,000 species, is the most species-rich genus in the family Cyperaceae, exhibiting a nearly worldwide distribution (Martín-Bravo et al. 2019; Plants of the World Online 2026). Recent phylogenetic studies have recognized six major lineages within *Carex*, namely the Siderostictae, Schoenoxiphium, Unispicate, Uncinia, Vignea, and core Carex clades, and proposed a revised infrageneric classification framework for the genus (Villaverde et al. 2020; Roalson et al. 2021). Under this framework, *Carex* has been classified into six subgenera, 62 formally named Linnaean sections, and 49 informal groups (Roalson et al. 2021). China represents one of the major centers of species diversity for *Carex*, with more than 600 species currently documented (Li and Zhang 2024).

Previous studies have confirmed that *Carex* subg. *Siderosticta* Waterway represents the earliest-diverging lineage of *Carex* and is sister to all remaining species of the genus (Waterway et al. 2009; Yano et al. 2014; Global Carex Group 2015, 2016; Starr et al. 2015; Villaverde et al. 2020). Species within subg. *Siderosticta* exhibit remarkable morphological diversity and are endemic to East and Southeast Asia, with a diversity center in the latter region (Roalson et al. 2021). The subgenus currently comprises 30 species traditionally assigned to four sections: sects. *Hemiscaposae* C.B.Clarke (12 species), *Siderostictae* Franch. (13 species), *Surculosae* Raymond (three species), and *Hypolytroides* T.Koyama (two species) (Roalson et al. 2021; Li et al. 2024).

Section *Hemiscaposae* has long been recognized as a distinct section within subg. *Vigneastra* (Tuck.) Kük (Dai et al. 2010). Following the recent description of a new species, the section recognized as such now comprises 12 species and three varieties, distributed mainly across south-central to southeastern China (including seven endemic species), as well as in Vietnam and Laos (Nguyen 2002; Dai et al. 2010; Ford et al. 2017; Yang and Chen 2005; Li et al. 2024). Morphologically, species of sect. *Hemiscaposae* are highly distinctive within *Carex*, characterized by the unique combination of lateral leafless culms, paniculate inflorescence, androgynous spikes, and remarkably broad, pseudopetiolate leaves reaching up to 12 cm in width (Dai et al. 2010; Ford et al. 2017). In addition, inflorescence architecture within the section is highly variable and has traditionally provided critical diagnostic characters for species delimitation (Ford et al. 2017).

During botanical field surveys in Hezhou City (Guangxi) and Jianghua County (Hunan), a distinctive species of *Carex* was collected. Through comprehensive morphological and micromorphological observations, molecular phylogenetic analyses, and examination of relevant herbarium specimens and literature, we determined it to be a new species belonging to the traditionally circumscribed sect. *Hemiscaposae* within subg. *Siderosticta*. Furthermore, during the examination of herbarium specimens, additional populations were discovered from Lianshan County (Guangdong). Herein, we describe and illustrate this species as new to science.

## Materials and methods

### Morphological comparison

Morphological observations of the new species were conducted based on both living plants collected in the field and specimens. The specimens of this new species were collected from Guangdong, Guangxi, and Hunan provinces, China. Nine vouchers from fieldwork of the new species were totally examined, and the morphological characters such as rhizomes, culms, leaves, bracts, spikes, glumes, utricles, and nutlets were observed and measured. We examined the relevant specimens of subg. *Siderosticta* (including types) deposited in E, GXMI, GXMG, HAST, IBK, IBSC, K, KUN, P, PE, WIN, and ZJFC, and compared the other specimens using online images from the Chinese Virtual Herbarium (https://www.cvh.ac.cn/) and JSTOR Global Plants (https://plants.jstor.org/).

### SEM observations on utricles and nutlets

We conducted scanning electron microscope (SEM) observations of the mature utricles and nutlets of the new species *Carex ensiformis* (collected from Jianghua, Hunan, *Z.C.Lu, W.B.Xu & J.Q.Huang LZC2929*, deposited in IBK), together with *C. damiaoshanensis* X.F.Jin & C.Z.Zheng (collected from Jiuwanshan National Nature Reserve, Guangxi, *S.Q.Chen 8873*, deposited in IBK). To remove residual silica from the nutlet surface, nutlets were soaked in a mixture of concentrated sulfuric acid and glacial acetic acid at a volumetric ratio of 1:9 for 12 h, rinsed three times with purified water, and subsequently treated in a bath-type ultrasonic cleaner with 70% ethanol for 7 min. The nutlets were air-dried at room temperature in the shade (Jin et al. 2014). The pretreated nutlets and clean utricles were mounted on stubs, coated with gold, and then observed and photographed under an SU8010 SEM (Hitachi, Tokyo, Japan).

### Sampling, DNA extraction, and sequencing

For molecular phylogenetic analyses, we sampled four individuals of *C. ensiformis* representing three populations from Hezhou and one from Jianghua (Table 1). Fresh leaves were dried in silica gel immediately after collection. Total genomic DNA was extracted using a modified CTAB method (Doyle and Doyle 1987). DNA concentration was measured using a fluorometer, and DNA integrity was assessed by electrophoresis on 1% agarose gels. Samples that passed quality assessment were used for library preparation. Deep genome skimming was subsequently conducted on the BGI DNBSEQ-T7 platform, generating 150 bp paired-end reads.

**Table 1:** Materials used deep genome skimming sequencing in this study, together with collection information. The data was deposited in the National Genomics data center (NGDC), BIG data center, Chinese Academy of Sciences, under project accession number PRJCA066294 (SAMC7885650–SAMC7885661). All vouchers were deposited in IBK and ZJFC respectively

Taxon	Accession number	Locality	Voucher
*Carex damiaoshanensis*	SAMC7885654	China, Guangxi, Rongshui	*Y. Qin & al. GXQY20210524032*
*C. densifimbriata*	SAMC7885656	China, Guangxi, Xing’an	*X. F. Jin 1044*
*C. densifimbriata* var. *hirsuta*	SAMC7885657	China, Guangxi, Longsheng	*Anonymous s.n.*
*Carex ensiformis* 1	SAMC7885650	China, Hunan, Jianghua	*Z. C. Lu & al. LZC2919*
*C. ensiformis* 2	SAMC7885651	China, Guangxi, Hezhou	*Z. C. Lu & al. LZC2942*
*C. ensiformis* 3	SAMC7885652	China, Guangxi, Hezhou	*Z. C. Lu & al. LZC2942*
*C. ensiformis* 4	SAMC7885653	China, Guangxi, Hezhou	*X. F. Jin 1030*
*C. lingii*	SAMC7885659	China, Fujian, Wuyishan	*Z. C. Lu LZC2947*
*C. scaposa* var. *dolicostachya*	SAMC7885661	China, Guangxi, Tianlin	*Z. C. Lu LZC1376*
*C. scaposa* var. *hirsuta*	SAMC7885660	China, Guangdong, Xinfeng	*Z. C. Lu & J. Q. Huang LZC2750*
*C. liouana*	SAMC7885655	China, Fujian, Yong’an	*Anonymous s.n.*
*C. ypsilandrifolia*	SAMC7885658	China, Fujian, Nanping	*Z. C. Lu LZC2974*

### Phylogenetic analysis

To capture the target phylogenetic fragments (ETS, ITS, *matK*, *ndhF*, and *rps16*) from the deep genome skimming data of the 12 newly sequenced samples, a combined assembly and local alignment strategy was deployed. First, the nuclear ribosomal DNA and plastid genomes were *de novo* assembled using GetOrganelle v1.7.7 (Jin et al. 2020) with default parameters. Subsequently, local BLAST databases were constructed for the assembled ribotypes and plastomes. Reference sequences containing the five target fragments were downloaded from GenBank and utilized as query sequences to perform local BLASTn searches against the constructed databases. Based on the optimal alignment positions and boundary coordinates, the exact homologous target sequences were precisely extracted using Samtools v1.22.1 (Li et al. 2009). These newly retrieved fragments, along with 74 sequences downloaded from NCBI, representing 29 samples and 26 species (including infraspecific taxa) were used for subsequent phylogenetic reconstruction (Tables 1 and 2). Finally, multiple sequence alignments for each marker were performed using MAFFT v7.525 (Nakamura et al. 2018) with L-INS-i strategy.

**Table 2:** Species with their GenBank accession numbers of DNA sequences used in the phylogenetic study. The – indicates that no sequence was available

Taxon	ITS	ETS	matK	ndhF	rps16
*Carex adrienii*	KP273628	KP273594	KP273663	KP273717	KP273771
*C. baccans*	KP273632	KP273598	KP273669	KP273723	KP273778
*C. cruciata*	KP273637	KP273603	KP273676	KP273730	KP273787
*C. geographica*	KX722473	–	KX722479	KX722485	KX722491
*C. glossostigma*	MN762656	–	KP273686	KP273740	–
*C. grandiligulata*	MW459022	MW458991	MW459089	–	–
*C. hypolytroides*	KP273647	KP273612	KP273690	KP273744	KP273800
*C. kucyniakii*	KP273651	KP273617	KP273695	KP273749	KP273805
*C. moupinensis*	KP273653	KP273619	KP273699	KP273753	KP273809
*C. pachygyna*	DQ998936	DQ998882	MW459090	–	–
*C. peliosanthifolia*	OR450685	OR463437	OR464515	OR464518	OR464521
*C. qingyuanensis*	OR450686	OR463436	OR464514	OR464517	OR464520
*C. scaposa*	OR450687	OR463438	OR464516	OR464519	OR464522
*C. siderosticta*	KP273658	KP273624	KJ513592	KJ513499	KP273817
*C. thinii*	KX722474	KX722468	KX722480	KX722486	KX722492
*C. tsiangii*	KU496610	KU377556	KU496590	–	–
*C. wuyishanensis*	MW459024	MW458993	MW459091	–	–

Following the classification of Roalson et al. (2021), *C. baccans* Nees and *C. cruciata* Wahlenb were selected as outgroups. The five aligned markers were subsequently concatenated into a single supermatrix, with each marker treated as a separate partition in the concatenated analyses. For the maximum likelihood (ML) analysis, the best-fit nucleotide substitution models for each partition were selected using ModelFinder (Kalyaanamoorthy et al. 2017) implemented in IQ-TREE under the Bayesian Information Criterion (BIC). The selected models were TPM2+F+G4 for ETS, TIM3+F+R2 for ITS, HKY+F+R2 for *matK*, HKY+F+I for *ndhF*, and TPM3+F for *rps16*. Maximum likelihood (ML) phylogenetic analyses were conducted using IQ-TREE v2.1.4 (Minh et al. 2020) with 1,000 ultrafast bootstrap (UFB) replicates (Hoang et al. 2018).

For the Bayesian inference (BI) analysis, the best-fit nucleotide substitution models for each gene region were selected using Akaike Information Criterion (AIC) in jModelTest v2.1.10 (Darriba et al. 2012). The selected models were GTR+G for ETS, ITS and *ndhF*, HKY+I for *matK*, and GTR+I for *rps16*. Bayesian inference (BI) phylogenetic analyses were conducted using MrBayes v3.2.7 (Ronquist et al. 2012) for 10,000,000 generations, sampling every 1,000 generations, with the first 25% of samples discarded as burn-in. The phylogenetic trees were visualized and modified using FigTree v1.4.4 (Rambaut 2018) and tvBOT (Xie et al. 2023).

## Results

### Morphological comparison

*Carex ensiformis* has culms lateral, basal leaves tufted, complex inflorescences with paniculate branches, spikes bisexual, androgynous, all arising from flowerless utriculiform cladoprophylls. These morphological characters are consistent with the traditional morphological circumscription of sect. *Hemiscaposae* (Dai et al. 2010). Morphologically, *C. ensiformis* is similar to *C. damiaoshanensis*, but differs in having a greater number of spikes, staminate and pistillate glumes smaller, utricles and nutlets smaller, and along with an extremely short beak at the utricle apex. Our phylogenetic results (see below) points that *C. ensiformis* is closely related to *C. densifimbriata* Tang & F.T.Wang, but differs in having narrower leaf blades, smaller utricles, and an extremely short beak of the utricle apex. A detailed morphological comparison of the three species is provided in Table 3.

**Table 3:** Morphological comparison among *C. ensiformis, C. damiaoshanensis*, and *C. densifimbriata*

Characters	C. ensiformis	C. damiaoshanensis	C. densifimbriata
Culms	20–55 cm tall	25–40 cm tall	30–80 cm tall
Leaf blades	Ribbon-like, ensiform or ribbon-oblanceolate, 1–2.5 cm wide	elliptic to elliptic-linear, 2–5 cm wide	elliptic to narrowly lanceolate, 2.8–4.5 cm wide
Pseudopetioles	from inconspicuous up to 20 cm long	6–10 cm long	2–20 cm long
Inflorescence branches	7–14	4–6	2–10
Spikes/each branched inflorescence	5–15	3 or 4	4–12
Staminate glumes	ovate	ovate-deltate	oblong-lanceolate
Pistillate glumes	ovate, 1–1.5 mm long	broadly ovate, 1.5–2 mm long	ovate-oblong, 2.8–3 mm long
Utricles	1.7–2.5 mm long; beak 0.2–0.5 mm long	4–5 mm long; beak ca. 2 mm long	3–3.5 mm long; beak 1.2–1.5 mm long
Nutlets	ovoid, ca. 1.5 mm long	obovoid, 2–2.5 mm long	ellipsoid, ca. 2 mm long

### Micromorphology of utricles and nutlets

**Fig. 1:**
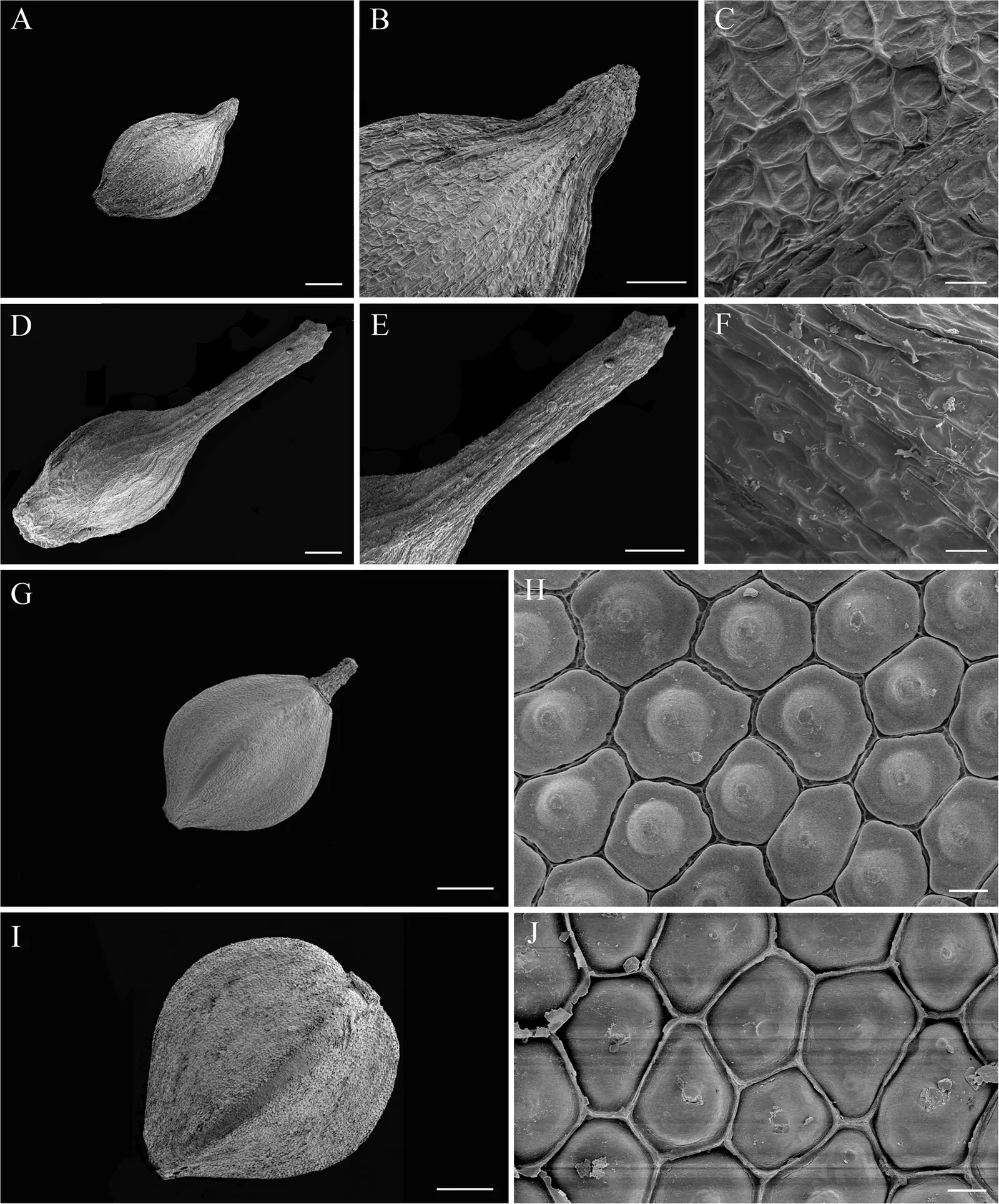
SEM micromorphology of utricles and nutlets of *Carex ensiformis* (**A–C**, **G**, **H**) and *C. damiaoshanensis* (**D–F**, **I**, **J**). **A** and **D** utricle overviews; **B** and **E** utricle beaks; **C** and **F** utricle surfaces; **G** and **I** nutlet overviews; **H** and **J** nutlet epidermal cells. Scale bars: 400 μm (**A**, **B**, **D**, **E**, **G**, **I**); 40 μm (**C, F**); 10 μm (**H, J**)

The micromorphology results of the utricles and nutlets by SEM are shown in Fig. 1.

The utricles of *Carex ensiformis* and *C. damiaoshanensis* are both ellipsoid, obtusely trigonous, glabrous, with many longitudinal veins; beak obliquely truncate with margins glabrous, but the utricle and beak lengths of *C. ensiformis* are significantly shorter than those of *C. damiaoshanensis*. The nutlet shape of *C. ensiformis* is ovoid, trigonous, and the one of *C. damiaoshanensis* is obovate, trigonous. The nutlet epidermal cells of both species are mainly irregular 5- or 6-gonal, with a large silica platform inside, and a single large silica body on its center. However, *C. ensiformis* differs from *C. damiaoshanensis* in having slightly undulate anticlinal cell walls (vs. straight). In addition, the utricle beak of *C. ensiformis* is erect and short, whereas that of *C. damiaoshanensis* is erect and distinctly longer.

### Molecular phylogenetics

**Fig. 2:**
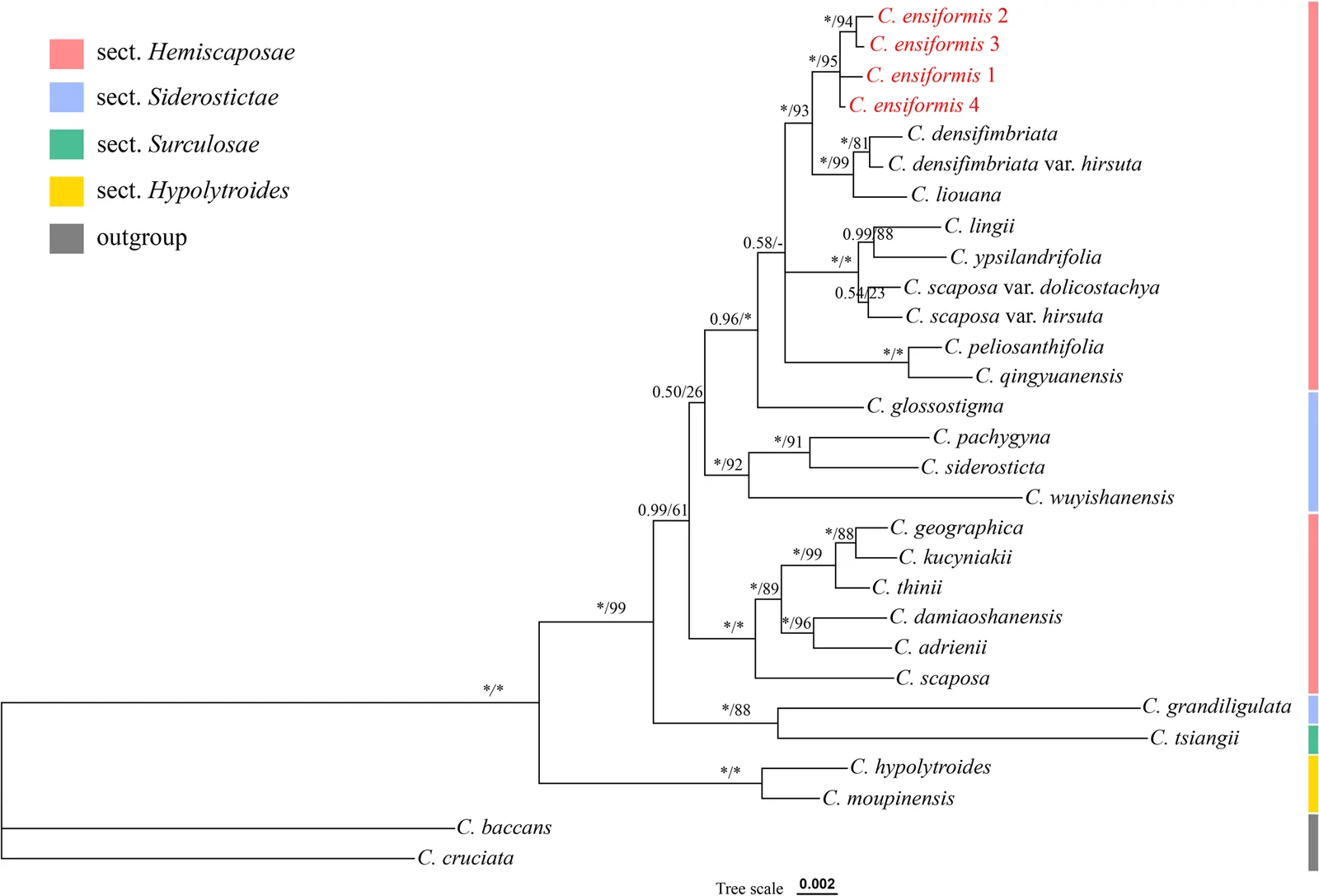
Bayesian phylogenetic tree reconstructed from the concatenated ETS, ITS, *matK, ndhF*, and *rps16* datasets. Bayesian posterior probabilities (PP) and ultrafast bootstrap support values (UFB, %) are indicated above the branches. Asterisks (*) indicate nodes with maximum support (PP = 1.00 and UFB = 100%). The new species is highlighted in red

The maximum likelihood (ML) and Bayesian inference (BI) trees reconstructed from the concatenated five-marker dataset produced largely congruent topologies, with the only topological incongruence involving the placement of *Carex glossostigma* Hand.-Mazz. (Fig. 2). The species of sect. *Siderostictae*, sect. *Hemiscaposae*, and sect. *Surculosae* formed a sister clade to the two species of sect. *Hypolytroides*, and all species within the sections received highly supported. Notably, both phylogenetic analyses consistently recovered the four individuals of the new species as a strongly supported monophyletic clade (PP = 1.00, UFB = 95%), which was resolved as sister to a clade comprising *C. densifimbriata*, *C. densifimbriata* var. *hirsuta* P.C.Li, and *C. liouana* F.T.Wang & Tang. Thus, the molecular phylogenetic results supported the distinctiveness of *C. ensiformis*, and indicated a closed relationship with these taxa. Section *Hemiscaposae*, as traditionally circumscribed taxon, was not recovered as monophyletic in either analysis. Therefore, the molecular placement of *C. ensiformis* should be distinguished from its assignment to sect. *Hemiscaposae* based on the traditional morphological circumscription.

## Discussion

*Carex* sect. *Hemiscaposae* has traditionally been recognized as a morphologically distinctive section within subg. *Indocarex* Ball. or subg. *Vigneastra*, characterizing by lateral culms, tufted basal leaves, paniculate inflorescences, bisexual androgynous spikes, and spikes arising from flowerless utriculiform cladoprophylls (Kükenthal 1909; Dai et al. 2000, 2010; Ford et al. 2017). *Carex ensiformis* possesses all of these diagnostic characters and can therefore be assigned to sect. *Hemiscaposae* based on morphological characters, with the aim of comparing it with the most similar related species. However, the monophyly of sect. *Hemiscaposae* has not been consistently supported by previous molecular phylogenetic studies, and our analyses based on five molecular markers likewise failed to recover the section as a monophyletic group. Although *C. ensiformis* was recovered within a well-supported monophyletic clade comprising several species traditionally assigned to sect. *Hemiscaposae*, demonstrating strong congruence between morphological and molecular evidence and supporting its placement among species of the section. This result should be interpreted as supporting *C. ensiformis* affinity with the section rather than demonstrating the monophyly of the section itself. These findings highlight the need for further taxon sampling and additional molecular data to clarify the phylogenetic limits and relationships of sect. *Hemiscaposae*. As the earliest-diverging lineage within *Carex*, subg. *Siderosticta* represents a key lineage for understanding the early evolutionary history of the genus (Waterway et al. 2009; Yano et al. 2014; Villaverde et al. 2020). The discovery of *C. ensiformis* not only enriches the species diversity of the subgenus, but also provides additional molecular evidence for resolving phylogenetic relationships within sect. *Hemiscaposae*.

Reproductive characters, particularly those of the utricle and nutlet, have long been regarded as among the most taxonomically informative characters for species delimitation in *Carex* (Kükenthal 1909; Jin et al. 2014; Jiménez-Mejías et al. 2016). Comparative morphological and SEM observations revealed that *C. ensiformis* differs from its morphologically similar species in utricle size, beak length, nutlet morphology, and the configuration of the anticlinal walls of the nutlet epidermal cells. These micromorphological differences provide additional diagnostic evidence for distinguishing the two species, and demonstrate that utricle and nutlet micromorphology can serve as a valuable complement to conventional macromorphological characters in the delimitation of closely related taxa.

Phylogenetic analyses recovered *Carex ensiformis* as the sister species of *C. densifimbriata*, although the two species are readily distinguishable morphologically. Such incongruence between phylogenetic affinity and morphological similarity has been reported in other *Carex* group (e.g., Stone and Wessinger 2024; Yan et al. 2025), suggesting that some morphological characters may not accurately reflect evolutionary relationships. Vegetative traits, such as culm height, leaf morphology, and inflorescence size, are more likely to be influenced by convergent evolution or phenotypic plasticity and therefore may provide limited resolution for species delimitation (Jiménez-Mejías et al. 2016). In contrast, reproductive characters, particularly those of the utricle and nutlet, are generally more conserved and consequently have greater taxonomic value for species delimitation.

Delimiting closely related species remains one of the major challenges in taxonomy, particularly in species-rich groups such as *Carex*, where extensive morphological plasticity and recent radiations frequently obscure species boundaries (Clerc-Blain et al. 2010; Jiménez-Mejías et al. 2016; Lu et al. 2021). With the rapid development of molecular phylogenetics, the infrageneric classification of *Carex* has undergone substantial revision. Increasing evidence has demonstrated that many traditional subgenera and sections established primarily on the basis of a limited number of morphological characters do not accurately reflect phylogenetic relationships, whereas molecular data provide a more robust framework for reconstructing evolutionary relationships within the genus (Global Carex Group 2015; Villaverde et al. 2020; Roalson et al. 2021). By integrating macromorphological, micromorphological, and molecular phylogenetic evidence, the present study supports the recognition of *C. ensiformis* as a distinct species, and further highlights the value of an integrative taxonomic approach for resolving species boundaries in *Carex* (Míguez et al. 2018; Luceño et al. 2021).

The discovery of *Carex ensiformis* as a new species within subg. *Siderosticta* not only enriches the species diversity of sect. *Hemiscaposae*, but also suggests that the diversity of *Carex* in southern China remains incompletely documented. Broader geographic sampling combined with genome-scale data will provide a more comprehensive understanding of phylogenetic relationships and morphological evolution within subg. *Siderosticta*.

## Conclusion

Based on morphological observations and molecular phylogenetic analyses, *Carex ensiformis* is herein described as a new species within subg. *Siderosticta*. The new species shares several characters with taxa traditionally placed in sect. *Hemiscaposae*, including lateral culms, compound paniculate inflorescences, numerous spikes with staminate flowers positioned above pistillate flowers, and utriculiform cladoprophylls. *C. ensiformis* is similar to *C. damiaoshanensis* and *C. densifimbriata*, but can be easily distinguished by its smaller staminate and pistillate glumes, smaller utricles and nutlets, and extremely short beak of the utricles.

The discovery of *Carex ensiformis* not only enriches the species diversity of *Carex* in southern China, but also further highlights the remarkable morphological diversity and taxonomic complexity within subg. *Siderosticta*. Furthermore, this study underscores the importance of continued botanical exploration and integrative taxonomic approaches in southern China, where additional cryptic or narrowly distributed species may still remain undiscovered.

Both morphological and molecular evidence strongly support *Carex ensiformis* as an independent species. Hereby we proceed to formally describe the new species below.

**Fig. 3:**
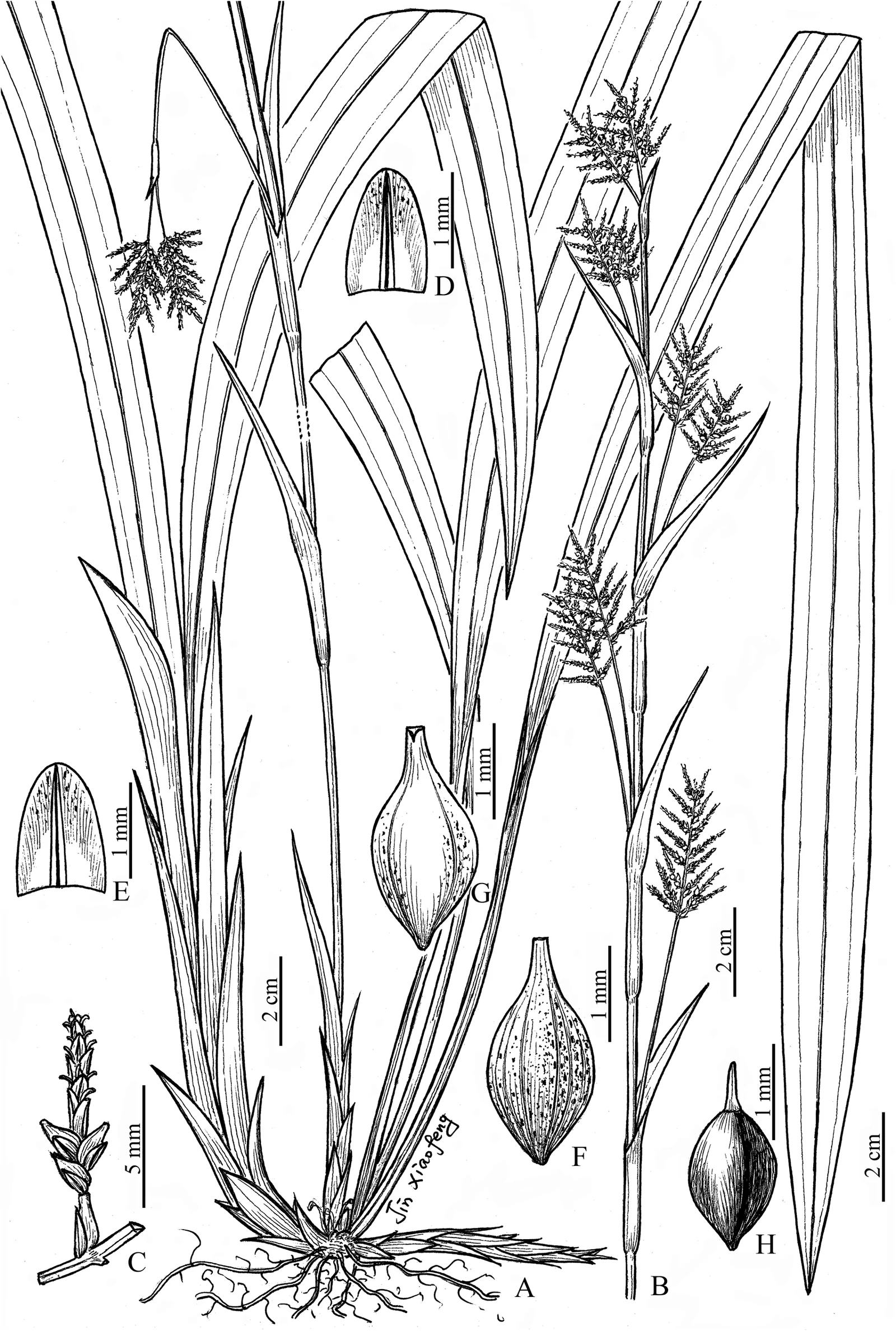
Line drawing of *Carex ensiformis.***A** habit; **B** inflorescence; **C** spike; **D** staminate glume; **E** pistillate glume; **F** utricle (dorsal view); **G** utricle (ventral view); **H** nutlet (drawn by Xiao-Feng Jin; based on the holotype: *Z. C. Lu et al. LZC2929* in IBK)

**Fig. 4:**
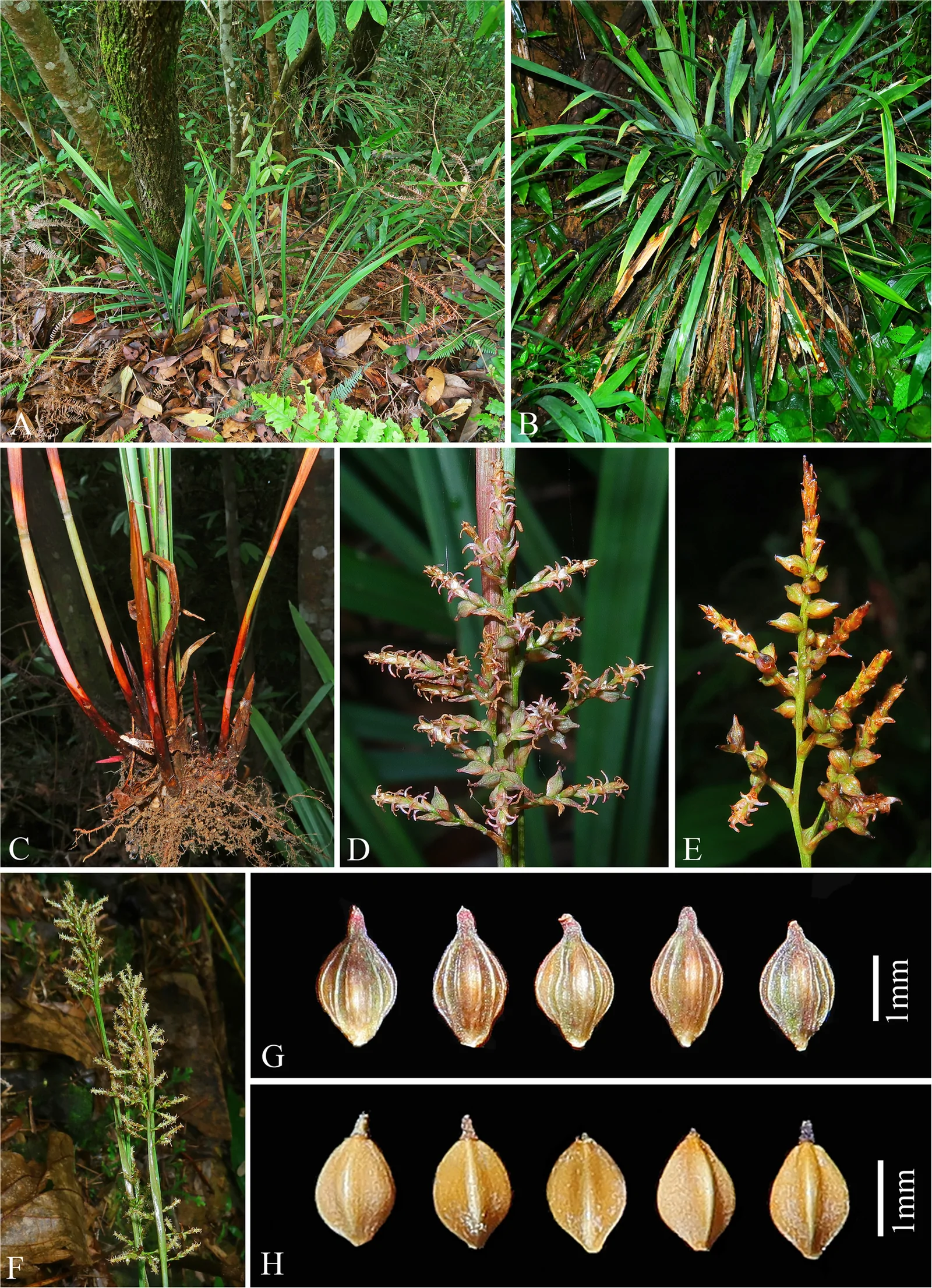
Morphological features and habitat of *Carex ensiformis.***A** habitat; **B** habit; **C** rhizome; **D** and **E** inflorescence branches showing spikes; **F** inflorescences; **G** utricles; **H** nutlets

*Carex ensiformis* Z.C.Lu, Y.F.Lu & X.F.Jin, sp. nov. Figures 1A–C, G, H; Figs. 3 and 4.

**Diagnosis** This new species is similar to *Carex damiaoshanensis* X.F.Jin & C.Z.Zheng (Fig. 1 D–F, I, J), but differs in having 7–14 inflorescence branches (vs. 4–6 branches), each branched inflorescence 5–15-spiked (vs. 3 or 4-spiked), utricles 1.7–2.5 mm long (vs. 4–5 mm long), apex abruptly contracted into a 0.2–0.5 mm long beak (vs. ca. 2 mm long beak), and nutlets ovoid, ca. 1.5 mm long (vs. obovoid, 2–2.5 mm long).

**Type:** CHINA. Hunan: Jianghua County, Weizhukou Township, Weizhuyuan, 111.774928°E, 24.836716°N, alt. 1050 m, 4 May 2025, *Z. C. Lu, W. B. Xu & J. Q. Huang LZC2929* (holotype: IBK barcode 00473476; isotypes: IBK barcode 00473477, PE and ZJFC barcode 00088895).

**Description** Rhizome woody, short, stoloniferous. Culms lateral, 20–55 cm tall, 1–3 mm in diameter, trigonous, glabrous, base with leafless pale brown sheaths. Leaves basal and cauline; basal leaves tufted, longer or shorter than culms, 20–40 × 1–2.5 cm, ribbon-like, ensiform or ribbon-oblanceolate, glabrous on both surfaces, or abaxially scabrid, base attenuate and decurrent along the petiole, apex acuminate or acute, margins entire, with three raised veins and many fine veins; pseudopetiole inconspicuous or up to 20 cm long, slightly flattened, glabrous; cauline leaves brown, spathe-like, papery, glabrous. Bracts spathe-like, shorter than branch inflorescences. Panicle compound, 7–14 branched; branch inflorescences paniculate, single or binate, rarely three, triangular-ovate, 1.2–2 × 1–2 cm; each branched inflorescence 5–15-spiked, base with a 1–7 cm long glabrous peduncles; inflorescence axes sharply trigonous; bractlets glumelike, narrowly ovate or lanceolate, brownish white, with deep brown spots, 2–2.5 mm long, glabrous. Spikes bisexual, androgynous, arising from flowerless utriculiform cladoprophylls, patent, 5–10 mm long; staminate part linear-cylindrical, 3–5 mm long, longer to shorter than pistillate part, 4–8-flowered; pistillate part 2–4 mm long, 4–8-flowered. Staminate glumes 1–1.5 mm long, pale brown, ovate, membranous, glabrous, apex with purple-red punctate lines. Pistillate glumes brown laterally, ovate, 1–1.5 mm long, membranous, glabrous, apex with purple-red punctate lines. Utricles pale yellow-brown, densely brown spotted, ellipsoid, obtusely trigonous, 1.7–2.5 mm long (including beak), papery, abaxially with 2 lateral veins, glabrous, subsessile, apex abruptly contracted into a 0.2–0.5 (0.7) mm long beak, orifice obliquely truncate. Nutlets brown at maturity, ovoid, trigonous, ca. 1.5 mm long; style base not or slightly thickened; stigmas 3.

**Etymology** The specific epithet ‘*ensiformis*’ refers to its ensiform leaves.

**Phenology** Flowering and fruiting from late March to early May.

**Additional specimens examined (paratypes) CHINA. Guangxi**: Lingui County (Lingchuan County now), Chaotian Township, Baishui Mountain, 21 March 1953, *L. Q. Chen 94496* (IBK, IBSC); Lingui County (Lingchuan County now), Chaotian Township, Qifen Mountain, 27 March 1953, *L. Q. Chen 94576* (IBK, IBSC); Hezhou City, Gupo Mountain, 23 April 2004, *X. F. Jin 1030* (ZJFC); Hezhou City, Babu District, Daning Town, Liwu Village, Huangliantang, alt. 770 m, 5 May 2025, *Z. C. Lu, W. B. Xu & J. Q. Huang LZC2942* (IBK, KUN, PE). **Hunan**: Jianghua County, Shuikou Town Degui Village to Dongtian Town Wujiang Village, 9 April 2016, *D. G. Zhang JH169* (JIU); Jianghua County, Weizhukou Township, Weizhuyuan, alt. 820 m, 4 May 2025, *Z. C. Lu, W. B. Xu & J. Q. Huang LZC2919* (IBK, CSH, HITBC); Jianghua County, Weizhukou Township, Weizhuyuan, alt. 1050 m, 4 May 2025, *Z. C. Lu, W. B. Xu & J. Q. Huang LZC2925* (IBK). **Guangdong**: Lianshan County, Hedong Town, Wanghou Mountain, *Nanling Exped. 487* (IBSC).

**Distribution and habitat** Based on field investigations and examination of herbarium specimens, *Carex ensiformis* is mainly distributed in northern Guangxi, northern Guangdong, and southern Hunan, China, whereas *C. damiaoshanensis* is currently known only from the Damiao Mountain region of Guangxi. *Carex ensiformis* grows sporadically in forests on mountain slopes, at an elevation of 750–1050 m.

**Conservation status** The new species has been recorded from two localities in Lingchuan County and two localities in Hezhou City, Guangxi; two localities in Jianghua County, Hunan; and one locality in Lianshan County, Guangdong. Its extent of occurrence (EOO) is estimated to be approximately 4,000 km^2^, which is a threshold for Vulnerable under criterion B1. However, *C. ensiformis* does not meet the additional conditions required for a threatened category under criterion B1: (a) the known populations occur in relatively continuous and well-preserved high-elevation forest habitats and are not considered severely fragmented; (b) there is no evidence of a continuing decline in EOO, area of occupancy (AOO), habitat quality, or the number of mature individuals; and (c) no extreme fluctuations in the population or distributional parameters have been observed. Furthermore, there is no evidence of a population reduction meeting the thresholds under criterion A, a small population size under criterion C, a very small or restricted population under criterion D, or a quantitative analysis indicating a high risk of extinction under criterion E. Therefore, based on the currently available distributional, population, and habitat information, *C. ensiformis* is assessed as Least Concern (LC) following the IUCN Red List Categories and Criteria (IUCN 2024).

## Data Availability

All data supporting the findings of this study are included in the main text. Newly generated sequences have been deposited in the National Genomics Data Center (NGDC), BIG Data Center, Chinese Academy of Sciences.
